# Efficacy of Sacroiliac Joint Injection With Anesthetic and Corticosteroid: A Prospective Observational Study

**DOI:** 10.7759/cureus.24039

**Published:** 2022-04-11

**Authors:** Siti Nur Fudhlana Ab Aziz, Zamzuri Zakaria Mohamad, Rajandra Kumar Karupiah, Aminudin Che Ahmad, Ahmad Sabri Omar

**Affiliations:** 1 Orthopedics, Hospital Tengku Ampuan Afzan, Kuantan, MYS; 2 Orthopedics, Traumatology and Rehabilitation, International Islamic University Malaysia, Kuantan, MYS; 3 Orthopedics, Traumatology and Rehabilitation, Kulliyyah of Medicine, International Islamic University Malaysia, Kuantan, MYS; 4 Orthopedics, Sultan Ahmad Shah Medical Centre (SASMEC) International Islamic University Malaysia, Kuantan, MYS; 5 Orthopedics, Hospital Raja Perempuan Zainab II, Kota Bharu, MYS

**Keywords:** low back pain, anesthetic, corticosteroid, sacroiliitis, sacroiliac joint

## Abstract

Sacroiliac joint injection aims to provide pain relief, improve work status, and early return to work. We aimed to investigate the role of corticosteroid and anesthetic mixture to provide short- and long-term pain relief in patients with sacroiliac joint pain. This prospective observational study included 27 patients with sacroiliac joint dysfunction who received a combination of triamcinolone and ropivacaine for sacroiliac joint injection followed by three scheduled visits at four weeks, eight weeks, and six months. The pain was assessed using visual analogue scale (VAS), physician's assessment on the number of positive provocative tests, and patients' self-reported assessment to evaluate their functional outcome. At the four weeks and eight weeks follow up evaluations, the mean VAS reduced from 5.85 (±1.03) at baseline to 3.30 (±1.77) at four weeks, 3.30 (±1.86) at eight weeks, and 3.00 (±1.86) at six months. At each interval, improvement in terms of clinical assessment using a series of provocative tests was seen with a mean of 1.37 (±1.33), 1.63 (±1.31), and 1.48 (±1.05) at four weeks, eight weeks, and six months, respectively. For the functional effectiveness parameters (Roland-Morris questionnaires), the sacroiliac joint (SIJ) injected with these drugs combination showed a more significant improvement in symptoms and function, baseline (13.56±3.36), at four weeks (9.04±3.33), at eight weeks (9.07±4.13), and six months (8.26±4.92). Using the one-way repeated measures ANOVA, the SIJ pain, provocative test, and functional outcome significantly improved over time after injection with triamcinolone and ropivacaine (p<0.001). No complications of the administration of these medications were noted. Our findings support the intermediate-term (six months) effectiveness and safety of a combination of corticosteroid and anesthetic injection for patients with SIJ dysfunction who failed conservative treatment.

## Introduction

Low back pain (LBP) is a common musculoskeletal condition. The incidence of low back pain is reported to be between 10% and 63%, with a median of 37% in several studies. In a suburban community survey in Malaysia, the incidence of low back pain was around 12%. There was a higher prevalence of approximately 60% in the population at risk (commercial vehicle drivers) [1]. LBP may arise from multiple anatomical structures, such as muscle, intervertebral disc, fascia, and facet joint. Another common cause of LBP includes the sacroiliac joint (SIJ). It is estimated that around 10-38% of LBP cases originated from SIJ [2].

Pain around the SIJ causes work restrictions, reduction in productivity, and causing impairment in quality of life. A presumptive diagnosis of SIJ pain can be made by clinical history, physical examination, and radiological test, as there is no standard test available to diagnose this pathology [3-6].

Treatment of SIJ pain includes conservative treatment, which consists of physiotherapy [2], manipulative therapy [7], anti-inflammatory agent, and ice [3]. Patients who fail this conservative treatment often require periarticular or intra-articular corticosteroid for pain relief [1,3]. The main goal of the sacroiliac joint injection is to provide pain relief, improve work status, and early return to work or pre-pain functional state [8].

Sacroiliac joint (SIJ) injection has proved effective in providing short-term pain relief in managing SIJ pain [9]. Our current observation aims to investigate the role of corticosteroid and anesthetic mixture to provide short- (less than eight weeks) and intermediate (six months) term pain relief in patients with SIJ pain. This study aimed to offer physicians simple guidance on the alternative therapeutic approach in managing SIJ pain.

## Materials and methods

This prospective observational study was conducted at Hospital Perempuan Zainab II, Kelantan, Malaysia, from June 2018 to July 2020. The patients were recruited from the outpatient spine clinic. Patients aged between 18 years and 70 years old diagnosed with sacroiliac joint dysfunction based on their history, clinical examination, and radiological findings were included in this study. Other inclusion criteria include chronic pain at the sacroiliac joint for more than 12 weeks, with a pain score of four or more on the visual analogue score (VAS). Our patients received NSAIDs and other analgesics and physiotherapy as pre-treatment but failed to achieve the desired level of pain control. Therefore, we can conclude that the conservative treatment failed in these patients, and SIJ injection can be helpful to elevate the pain. Patients with coagulation disorder, connective tissue disorder, and pain due to infective origin were excluded from this study. Participation was voluntary. All the participants gave their informed consent. The National Medical Research Register (NMRR) and Medical Research and Ethics Committee (MREC) approved this study (ID number: NMRR-18-2676-43284).

The patient underwent pre-injection clinical evaluation. Collected patient demographic data included age, gender, height, and weight. The intensity of SIJ pain felt is recorded using a visual analogue score (VAS), presented by a 10 cm horizontal line representing the patient's pain intensity. Zero represents "no pain" while the upper limit (10 cm) represents the "worst pain imaginable" [10]. A difference of two scores in VAS assessment, as suggested by Danoff et al. in his study, is considered a reasonable threshold to identify successful pain intervention, therefore, adopted in this current study [11].

The clinical evaluation includes pain or local tenderness at the affected SIJ, flexion, abduction, and external rotation (FABER) test, Gaenslen's and Fortin finger test [12,13]. These tests are provocative tests that will reproduce pain at the affected SIJ. FABER test (also known as Patrick's test) is performed in a supine position by placing the affected hip in 90° flexion, abduction, and external rotation, with the ankle of the affected limb resting on the contralateral knee, creating a figure of four. Downward pressure is applied to the knee of the affected limb. The test was considered positive when pain reproduced along the SIJ. Gaenslen's test was also performed in a supine position, where passive flexion of hip and knee at the non-affected side, while the affected limb is allowed to fall to the side of examination table gradually. The test is considered positive when the patient complains of pain in the affected joint (the hanging side leg). Fortin's finger test is where the patient was asked to point to the area of pain with one finger. The test is considered positive if the site is within 1 cm of the posterior iliac spine.

Patients were subsequently evaluated using a plain radiograph. X-rays were reviewed by a senior consultant and were graded based on the New York Classification [14]. Based on this classification, x-rays can be graded as grade I to IV - wherein grade I, x-ray can be normal; in grade II, there is localized sclerosis or erosion with no widening or narrowing of joint space. There is moderate joint erosion or minimal evidence of joint ankylosis in grade III with narrowing or widening of joint space. In grade IV, the joint appeared ankylosed in the x-ray. This study involved those patients with grades I and II x-rays.

Patients were also required to answer Roland-Morris Disability (RMD) questionnaire as a pre-injection functional status assessment. This questionnaire consists of 24 questions describing different movements and functions for which patients tick those that apply to them daily. This questionnaire aimed to identify physical problems and not psychological or social problems, therefore adopted in the study. Even though we are using the English version, this questionnaire is easy to understand and consists of yes or no answers - an explanation given whenever is needed. The total score is the number of questions with a positive response (yes). A reduction in four or more scores indicates that the treatment has achieved its goal [15].

The procedure is performed using fluoroscopy [16]. The SIJ injections are performed by a senior surgeon using a posterior approach with a standard single beam C-arm fluoroscopy to guide needle placement. The patient was placed in a prone position. Skin prepared before injection to maintain sterility of procedure. The skin is infiltrated with a local anesthetic before needle placement. A spine needle, size 22 G, 3.5 inches (Spinocan; B. Braun: Petaling Jaya, Malaysia), was used for the procedure. Needle tip navigated gradually towards the joint space using fluoroscopic guidance and aimed at the lower one-third of SIJ, approximately 1 cm from the distal part of SIJ. Contrast media 0.5-1.0 mL was injected to confirm the location of the needle. In intraarticular injection, contrast traveled along the joint. Contrary to periarticular injection, the contrast will accumulate in the periarticular space, which is visualized as local pooling of contrast at the injection sites during assessment with fluoroscopy.

Our study included intra- and periarticular injections as both techniques have proven effective in treating sacroiliac joint pain [15]. Next, a 2 mL mixture of ropivacaine (0.2%) and triamcinolone 40 mg was injected. The anesthetic has a diagnostic and treatment effect in treating pain around SIJ [2]. It provides the initial pain control, while corticosteroids provide more extended pain relief. Following the procedure, the patient is monitored in the recovery area. A simple oral analgesic is prescribed post-injection.

Post-injection evaluation was performed at four weeks, eight weeks, and six months. The primary outcome variable was a reduction in pain scores. The second variables' efficacy measures include the number of positive provocative tests to reproduce pain at the affected SIJ and RMD questionnaires measures during visits.

All the data (clinical and radiological) were entered into a computerized database. For a continuous variable, a one-way repeated measures ANOVA was conducted to compare pain scores post-SIJ injection at baseline (before injection) and follow-up at four weeks, eight weeks, and six months. Data were analyzed using a one-way repeated measure ANOVA test after its normal distribution was confirmed. All assumptions of the test were also checked and met. P-values of less than 0.05 are considered statistically significant. Data were presented in mean and standard deviation for numerical data; frequencies and percentages were used for categorical data.

## Results

There were a total of 31 patients involved in this study. Twenty-seven patients completed the six months follow-up. Four patients did not complete the six months follow-up, which was considered a protocol violation; therefore, the patients were excluded from the analysis. Of the 27 patients, 20 patients received unilateral SIJ injection (in nine {33.3%} patients procedures were performed on right SIJ, and in 11 {40.7%} patients on the left) and seven (25%) patients received SIJ injection on bilateral sit (Table 1).

**Table 1:** Sociodemographic of patients (n=27).

Variable		Mean	SD	n	%
Age		46.63	11.31	-	-
Height		1.57	0.10	-	-
Weight		64.08	16.51	-	-
Gender	Male	-	-	5	18.5
	Female	-	-	22	81.5
Injection site	Right	-	-	9	33.3
	Left	-	-	11	40.7
	Both	-	-	7	25.9
X-ray grade	I	-	-	20	74.1
	II	-	-	7	25.9

Following the SIJ injection, pain relief was observed at each of the three visits - 85% (n=23) experienced pain relief at four weeks, 81% (n=22) at eight weeks, and 74% (n=20) at six months. Recurrence of pain was observed in five patients (three at eight weeks and two at six months). The mean pain score pre-treatment was 5.85 ± 1.03, the mean pain score at four weeks was 3.30 ± 1.77, at eight weeks was 3.30 ± 1.86, and at six months was 3.0 ± 1.86, therefore it shows a significant reduction as compared to baseline (p<0.001). Continuous assessment with repeated one-way ANOVA shows significant changes in pain score over time (p<0.001) (Table 2).

**Table 2:** Means of VAS score (cm) over time (n=27). One-way repeated measures ANOVA show significant changes in pain score over time (p<0.001). *Post-hoc test with Bonferroni adjustment shows a significant difference between baseline and follow-up at our weeks, baseline and follow-up at eight weeks, and baseline and follow-up at six months (p<0.05). VAS: visual analogue scale

Timepoint	n	Mean (SD)	Change from baseline (SD)	p-Value
Baseline	27	5.85 (1.03)*	-	-
Follow-up at four weeks	27	3.30 (1.77)*	-2.56 (1.28)	<0.001
Follow-up at eight weeks	27	3.30 (1.86)*	-2.56 (1.67)	<0.001
Follow-up at six months	27	3.00 (1.86)*	-2.85 (1.56)	<0.001

Similar trends following SIJ injection were observed in other variables at each interval compared to baseline. After four weeks following SIJ injection, the change in the provocative test from baseline was significantly appreciable (p<0.001). In addition, a trend toward more remarkable improvement in clinical assessments was observed at eight weeks (p<0.002) and six months (p<0.001) as compared to baseline.

**Table 3:** Mean of positive provocative test over time (n=27). One-way repeated measures ANOVA show significant changes in positive provocative test over time (p<0.001). *Post-hoc test with Bonferroni adjustment shows a significant difference between baseline and follow-up at four weeks, baseline and follow-up at eight weeks, and baseline and follow-up at six months (p<0.05).

Timepoint	n	Mean (SD)	Change from baseline (SD)	p-Value
Baseline	27	2.78 (1.07)*	-	-
Follow-up at four weeks	27	1.37 (1.33)*	-1.41 (1.28)	<0.001
Follow-up at eight weeks	27	1.63 (1.31)*	-1.15 (1.70)	<0.002
Follow-up at six months	27	1.48 (1.05)*	-1.30 (1.49)	<0.001

The mean RMD score of the 27 patients pre-treatment was 13.56 ± 3.36, while on the subsequent follow-up at four weeks was 9.04 ± 3.36, at eight weeks was 9.07 ± 4.13, and at six months was 8.26 ± 4.92. These scores show significant improvement at each follow-up interval (p<0.001). The continuous analysis also shows a significant effect of time on the provocative test and RMD score with p<0.001 (Tables 3, 4).

**Table 4:** Means of Rolland Morris disability score over time (n=27). One-way repeated measures ANOVA show significant changes in the Roland Morris disability score over time (p<0.001). *Post-hoc test with Bonferroni adjustment shows a significant difference between baseline and follow-up at four weeks, baseline and follow-up at eight weeks, and baseline and follow-up at six months (p<0.05).

Timepoint	n	Mean (SD)	RM score change from baseline (SD)	p-Value
Baseline	27	13.56 (3.36)*	-	-
Follow-up at four weeks	27	9.04 (3.33)*	-4.52 (2.89)	<0.001
Follow-up at eight weeks	27	9.07 (4.13)*	-4.48 (3.91)	<0.001
Follow-up at six months	27	8.26 (4.92)*	-5.30 (4.07)	<0.001

This observation concluded that the intervention over six months elicits a statistically significant reduction in clinical and patient functional status following SIJ injection. This procedure has been shown to pose potential complications such as infection and possible post-injection flare. No complication occurred in our case.

## Discussion

Corticosteroid injection in the joints is widely used to treat various arthritis. Sacroiliac joint injection efficacy is usually rapid and short-lived. Initial studies show short-term pain relief with a minimum duration of one month [17,18]. However, the studies do not provide sufficient data for durations of pain relief and do not provide validated outcome measures.

In the current study, we were able to demonstrate that SIJ patients who received a mixture of anesthetic and corticosteroid treatment had an improvement in pain, especially at four weeks of follow-up (p<0.001), which is similar to a previous study by Luukainen et al. [19]. Another study by Karabacakoglu et al. supported these findings [18]. Our study includes a longer follow-up, therefore enabling us to obtain further information on the duration of effectiveness of treatment. From our continuous observation, pain shows significant improvement at eight weeks and six months (p<0.001).

In an earlier study by Maugars et al., the efficiency of SIJ injection shows 85.7% improvement at one month following injection, 62% at three months, and 58% at six months [20]. Their results are similar to us at one-month follow-up (85% of injected patients with improvement); however, our result at six months (74% of injected patients) shows a better outcome.

Most of the previous studies emphasize the intra-articular nature of SIJ injection compared to us, who accept both intra- and periarticular injection [17,18,20]. It is difficult to obtain intra-articular placement, especially in the presence of spondyloarthropathy [21]. As our outcome shows improvement in pain relief, it is safe to conclude that each technique is an acceptable and reliable method of treatment to reduce pain in SIJ.

Symptom description in sacroiliac joint pain can be dissimilar for each patient. Clinical observation of pain shows that the pain arising from the sacroiliac joint can be reproduced by provocative tests [22]. Reduction in provocative tests shows improvement in pain, thus may suggest that SIJ injection can be used to improve pain at the sacroiliac joint. Our current finding at one month is similar to studies done by Luukkainen et al. in 2002 and Maugars et al. in 1996, in which the patients who received corticosteroid injection for SIJ pain experience reduce in provocative stress tests [19,20]. Further observation in our study also shows a significant reduction in provocative tests at eight weeks (p<0.002) and six months (p<0.001).

The previous studies did not include the assessment for the functional outcome, which is more important in understanding the effect of injection on a patient's daily living. The functional status is one of the factors which can determine the successful treatment provided to the patient. Several tools are available to assess sacroiliac joint pain. This tool includes the visual analogue scale [19], Oswestry Disability Index [23], and Japanese Orthopedic Association (JOA) scoring system [16]. This self-reported assessment can consist of either physical or psychological factors or both.

In this study, we used Roland-Morris questionnaires to assess the functional status of patients receiving sacroiliac joint injections. The scoring system did not indicate the patients' pain directly but evaluated pain related to daily activity. From our findings, SIJ injection with anesthetic and corticosteroid mixture provide adequate pain relief and improve patient's function, which is shown by the decreased RMD score, which is significant up to six months. This finding is comparable to the previous study by Liliang et al., which shows significant pain improvement with an average observation period of 45.4 weeks [24].

This prospective observational study is conducted in a single center that reflects actual population clinical practice. This study, however, did not have a control group, thus may pose selection bias. The lack of a comparison group in this current study can be explained in several ways. First, recruitment was difficult. Secondly, due to ethical issues, depriving patients of receiving appropriate treatment when the treatment is accessible can cause unnecessary pain, disturbing their daily living.

Informed consent and patients who were not easily convinced of the benefit of this study posed a hurdle in recruitment. Further study with larger sample size, different study designs with a control group, and randomization, followed by a longer follow-up duration, may be required to validate these findings.

## Conclusions

Our findings demonstrate pain relief following SIJ injection with a combination of corticosteroid and anesthetic mixture in patients with SIJ pain. This improvement is significant for a short-term duration and, at the same time, shows a significantly longer-term positive response (up to six months) for patients satisfaction. Reduction in pain also indirectly improves a patient's functional status, as shown in our result. From this study, we think that SIJ injection is a reasonable approach to treating persistent SIJ pain. However, further randomized controlled trials can be designed to assess long-term side effects, disease progression, and the efficiency of this technique.
